# Low-molecular-mass secretome profiling identifies HMGA2 and MIF as prognostic biomarkers for oral cavity squamous cell carcinoma

**DOI:** 10.1038/srep11689

**Published:** 2015-07-03

**Authors:** Kai-Ping Chang, Shih-Jie Lin, Shiau-Chin Liu, Jui-Shan Yi, Kun-Yi Chien, Lang-Ming Chi, Huang-Kai Kao, Ying Liang, Yu-Tsun Lin, Yu-Sun Chang, Jau-Song Yu

**Affiliations:** 1 Departments of Otolaryngology-Head & Neck Surgery, Chang Gung Memorial Hospital, Tao-Yuan, Taiwan; 2 Graduate Institute of Biomedical Sciences, College of Medicine, Chang Gung University, Tao-Yuan, Taiwan; 3 Molecular Medicine Research Center, Chang Gung University, Tao-Yuan, Taiwan; 4 Department of Medical Research, Chang Gung Memorial Hospital, Tao-Yuan, Taiwan; 5 Department of Plastic & Reconstructive Surgery, Chang Gung Memorial Hospital, Tao-Yuan, Taiwan; 6 Department of Cell and Molecular Biology, College of Medicine, Chang Gung University, Tao-Yuan, Taiwan

## Abstract

The profiling of cancer cell secretomes is considered to be a good strategy for identifying cancer-related biomarkers, but few studies have focused on identifying low-molecular-mass (LMr) proteins (<15 kDa) in cancer cell secretomes. Here, we used tricine–SDS-gel-assisted fractionation and LC–MS/MS to systemically identify LMr proteins in the secretomes of five oral cavity squamous cell carcinoma (OSCC) cell lines. Cross-matching of these results with nine OSCC tissue transcriptome datasets allowed us to identify 33 LMr genes/proteins that were highly upregulated in OSCC tissues and secreted/released from OSCC cells. Immunohistochemistry and quantitative real-time PCR were used to verify the overexpression of two candidates, HMGA2 and MIF, in OSCC tissues. The overexpressions of both proteins were associated with cervical metastasis, perineural invasion, deeper tumor invasion, higher overall stage, and a poorer prognosis for post-treatment survival. Functional assays further revealed that both proteins promoted the migration and invasion of OSCC cell lines *in vitro*. Collectively, our data indicate that the tricine–SDS-gel/LC–MS/MS approach can be used to efficiently identify LMr proteins from OSCC cell secretomes, and suggest that HMGA2 and MIF could be potential tissue biomarkers for OSCC.

Oral cavity cancer is one of the common cancers worldwide, accounting for more than 10,000 deaths per year^1^,^2^. It can arise from various locations, including the tongue, buccal area, gingiva, lip, floor of mouth, and hard palate. Alcohol, tobacco, betel quid chewing and viral infections are the main risk factors for oral cavity cancer^3^,^4^,^5^,^6^,^7^. Most oral cavity cancers correspond to oral cavity squamous cell carcinomas (OSCCs), which are quite locally aggressive, and are characterized by a moderate locoregional recurrence rate and a poor survival rate^8^,^9^,^10^. Despite the use of improved treatment modalities, including surgery, radiotherapy and chemotherapy, the 5-year overall survival rate of OSCC patients is only ~ 60%^11^,^12^,^13^. To deepen our knowledge and improve the management of this common disease, we need a systemic approach to discover the carcinogenesis-related proteins in OSCC cells.

In a previous study, we identified metastasis-associated proteins by using laser microscopy to capture tumor cells from metastatic cervical lymph nodes and the corresponding primary tumor, labeling the extracted cellular proteins with iTRAQ tags (isobaric tags for relative and absolute quantitation), and performing comparative proteomic analyses^14^. We also established proteomic profiles from OSCC cell secretomes and compared them between primary tumors and adjacent non-tumor epithelia from OSCC patients^15^,^16^. Although these previous works offered systematic approaches for the proteomic profiling of OSCC during carcinogenesis and metastasis, these approaches missed some low-molecular-weight (LMr) proteins due to technical limitations: LMr proteins are less likely than larger molecules to be detected in complex biological mixtures subjected to routine proteomic profiling based on mass spectrometry (MS).

In an effort to discover novel OSCC biomarkers/therapeutic targets, we used tricine-SDS-gel-assisted fractionation in conjunction with liquid chromatography-tandem mass spectrometry (LC-MS/MS) to systematically identify LMr proteins in the secretomes of five OSCC cell lines. We then analyzed a number of OSCC tissue transcriptome databases available in the public domain, searching for proteins that are specifically overexpressed in OSCC tumor cells compared to the normal oral epithelium. By combining these approaches, we identified HMGA2 and MIF (which had not previously been uncovered as relevant proteins using the traditional approaches) as potential biomarkers and possible therapeutic targets for OSCC.

## Results

### Generation of the OSCC LMr secretome dataset

**Figure 1:**
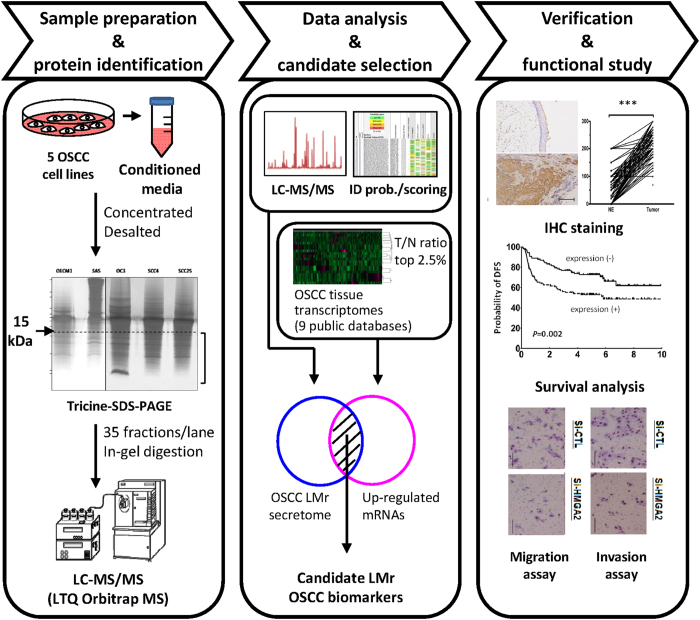
Workflow for the discovery of LMr OSCC biomarkers through the combined analysis of the low-molecular-mass OSCC cell secretomes and OSCC tissue transcriptomes.

Figure. 1 shows our strategy for identifying the OSCC LMr secretome and using it for biomarker discovery. Briefly, serum-free conditioned media from five OSCC cancer cell lines (OC3, OEC-M1, SAS, SCC4, and SCC25) were concentrated, desalted, and fractionated by tricine-SDS-PAGE, which can efficiently resolve proteins in the molecular mass range of 1 to 100 kDa^17^. The LMr region of the gel (<15 kDa) was excised, subjected to in-gel trypsin digestion, and analyzed with an LTQ-Orbitrap mass spectrometer. The Mascot program was used to search the MS spectra against the Swiss-Prot database, and the Scaffold software was to generate the LMr secretome dataset from the obtained results. We then compared the secretome dataset to the sets of genes found to be upregulated in the OSCC tissue transcriptome dataset (retrieved from the Gene Expression Omnibus or ArrayExpress database). Candidates that were present in the secretome dataset and upregulated in one or more of the OSCC transcriptome databases were designated as candidate LMr OSCC biomarkers. These candidate LMr OSCC biomarkers could represent a good reservoir for the identification of diagnostic biomarkers and/or therapeutic targets of OSCC.

**Figure 2:**
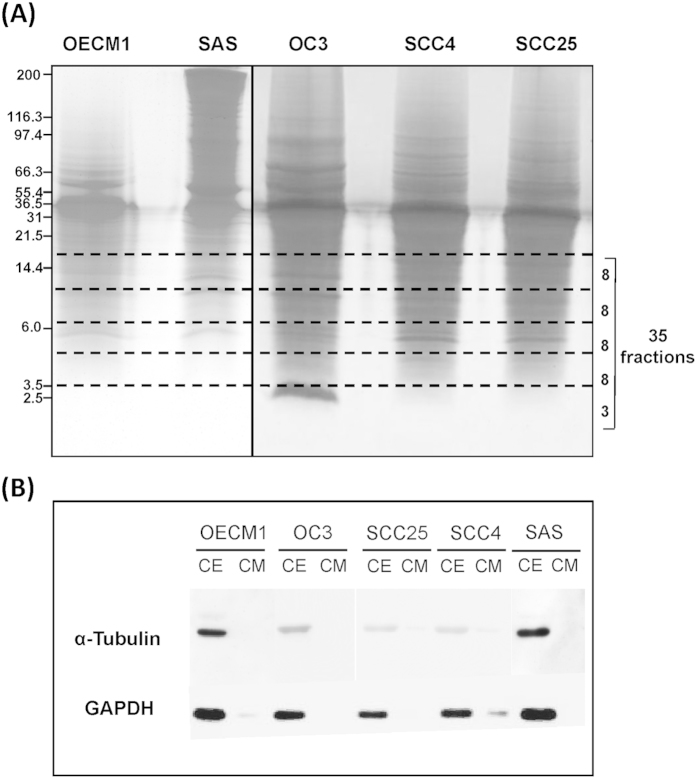
Separation and identification of LMr proteins from the conditioned media of five OSCC cell lines. (**A**) Proteins in conditioned media (500 μg) were separated by tricine-SDS-PAGE and visualized using Coomassie Brilliant Blue staining. The portion of each gel lane below 15 kDa was divided into 35 fractions, excised, and subjected to in-gel tryptic digestion. The digested peptides from each fraction were analyzed by LC-MS/MS. (**B**) Proteins (40 μg) from conditioned media (CM) and cell extracts (CE) of the OC3, OEC-M1, SAS, SCC4, and SCC25 cell lines were analyzed by Western blotting using antibodies against α-tubulin and GAPDH.

Conditioned media were obtained from cultures of the five OSCC cell lines, and the secreted proteins were separated on gels and subjected to protein staining (Fig. 2A). The gel fragments corresponding to each gel lane below 15 kDa were divided into 35 fractions, excised, subjected to in-gel tryptic digestion, and analyzed in triplicate by LC-MS/MS. For quality analysis, the conditioned media and cell lysates were subjected to Western blot analysis for the presence of two abundant cytoplasmic proteins: α-tubulin and glyceraldehyde-3-phosphate dehydrogenase (GAPDH). As shown in Figure. 2B, the two proteins were clearly detected in the cell extracts, but were barely detectable in the conditioned media. Consistent with the results of our previous study^18^, these results indicate that the proteins recovered in the conditioned media were not present in the media due to cell death.

**Figure 3:**
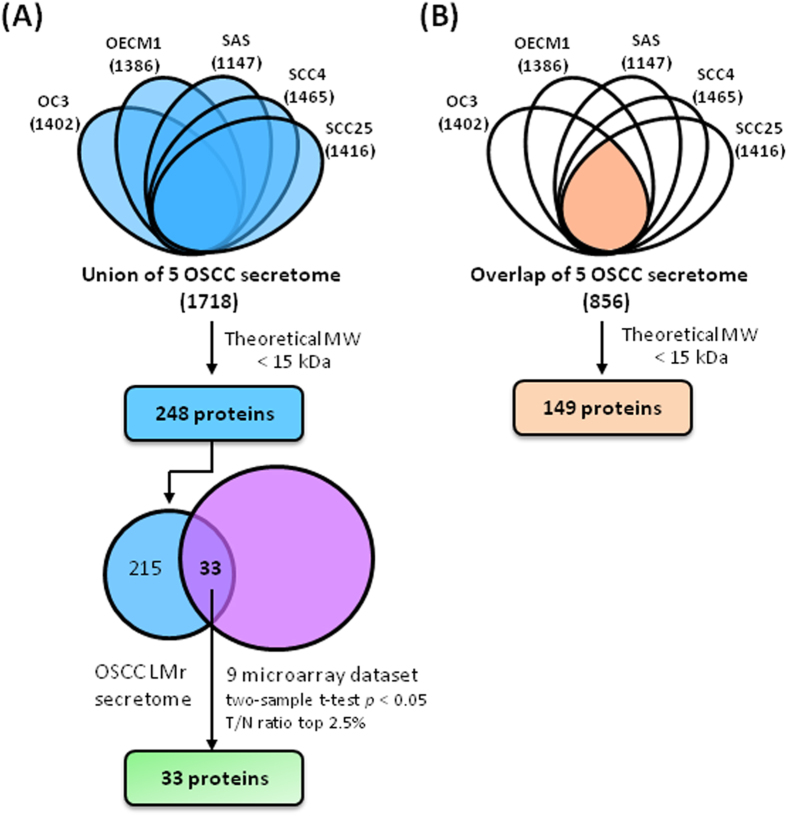
Informatics analysis flowchart for the discovery of LMr OSCC biomarkers. The LMr secretome profiles obtained from the five OSCC cancer cell lines were combined together to find proteins detected in any one of the five cell lines (**A**) or detected commonly in all five cell lines (**B**). The list of proteins generated in (**A**) was then processed to identify “true” LMr proteins and compare with OSCC tissue gene expression data available in the public domain (upregulated mRNAs) for the discovery of LMr OSCC biomarkers.

We analyzed the resulting MS and MS/MS spectra in combination with the results from triplicate LC-MS/MS runs using the appropriate software and criteria, and identified 1402, 1386, 1147, 1465 and 1416 proteins in the OC3, OEC-M1, SAS, SCC4 and SCC25 cell lines, respectively (Fig. 3A). Together, a total of 1718 nonredundant proteins were detected in the five OSCC LMr secretomes, as either intact proteins or degraded fragments (Table S1). Of them, 248 (14.4%) were found to have theoretical intact molecular masses less than 15 kDa, and were thus further classified as “true” LMr proteins (Table S2 and Fig. 3A). The five OSCC LMr secretomes had 856 proteins in common (Table S3), 149 of which were predicted to be true LMr proteins (Table S4 and Fig. 3B). The latter group (856 proteins) included numerous cytokines, chemokines, and growth factors that are usually present at very low concentrations in body fluids, such as BMP1, CXCL1, CXCL2, CXCL3, CXCL6, CXCL10, CXCL11, HDGF, IGFBP3, IGFBP4, IGFBP7, IGFL1, IGF2, IL1A, IL6, IL8, IL11, IL18, VEGFA, CTGF, TGFB1, VEGFC, TGFB2 and PDGFA (Table S1). Thus, the modified GeLC-MS/MS approach used herein not only identifies LMr proteins, it can also detect extremely low-abundance proteins that are known to have important biological functions in the extracellular space.

We herein identified 1718 potential LMr proteins in our initial secretome analysis, but subsequently found that only 14.4% (248/1718) appeared to be true LMr proteins. This suggests that ~85% (1470/1718) of the detected proteins may instead (despite the use of a protease inhibitor cocktail) represent the products of protein degradation taking place in the conditioned media. This could indicate that the degree of protein degradation in the concentrated secretome may profoundly affect the efficiency of our strategy for enriching and identifying true LMr proteins from the secretome. We therefore evaluated whether our strategy was more efficient for enriching/identifying the true OSCC LMr secretome, compared to the conventional GeLC-MS/MS strategy (i.e., resolution of the secretome on 8–14% gradient SDS gels) used in our previous study of 23 cancer cell lines^18^. Indeed, of the 1799 proteins previously identified in the secretomes from two OSCC cell lines (OCE-M1 and SCC-4), only 75 were LMr proteins^18^. This proportion is significantly lower than that observed in the present study (248 LMr proteins out of 1718 identified proteins) (Table S5).

We also analyzed LMr proteins (<15 kDa) identified in the secretomes of other cell lines. We selected these LMr proteins from 13 previous reports that had available molecular mass information and reported the identification of >500 proteins using conventional GeLC-MS/MS or in-solution digestion/LC-MS/MS strategies. This review revealed that the selected previous studies had identified 12 to 173 LMr proteins in the secretomes from a variety of cancer and non-tumor cell lines (starting with 20 μg to 1 mg of secreted proteins) (Table S5). For reference purposes, we also calculated the percentage of LMr proteins (<15 kDa) in the total human proteome from the Swiss-Prot database (released June 15, 2010; 20,306 entries), and found that this total human proteome contained 1715 LMr proteins (8.4%, 1715/20,306) (Table S5). These observations suggest that preparative tricine-SDS-PAGE coupled with LC-MS/MS is a simple and efficient strategy for identifying a greater number of LMr proteins in a cancer cell secretome.

### Secretion pathway prediction and ontology of the MS-identified LMr proteins

The 248 MS-identified LMr proteins were analyzed for their predicted protein secretion pathways. Of the identified, LMr proteins, 25 (10.08%), 150 (60.48%) and 7 (2.82%) were predicted to be released from OSCC cells by the signal peptide-, non-signal peptide- and membrane protein shedding-mediated secretion pathways, respectively (Figure S1 and Table S6). Thus, of the 248 MS-identified LMr proteins, 182 (73.38%) were predicted to be secreted/shed from OSCC cell lines via these three pathways.

Next, the 248 MS-identified LMr proteins were subjected to a Gene Ontology (GO) analysis using the Blast2GO software^19^. All of the identified LMr proteins corresponded to at least one annotation item in the GO categories of molecular functions, biological processes, and cellular components (Figure S2). The most common annotated molecular functions were cation binding (43 proteins), enzyme binding (36), receptor binding (36) and RNA binding (36). The main biological process classifications were cellular macromolecule metabolic processes (143 proteins), regulation of cellular processes (140), and cellular nitrogen compound metabolic processes (122). The three major cellular component categories included intracellular organelle proteins (199 proteins), cytoplasmic proteins (192) and cytoplasmic parts (168).

### Identification of potential LMr biomarkers of oral cancer via dataset integration

We next retrieved the gene expression profiles of nine OSCC tissue gene expression datasets (E-MEXP-44, E-TABM-302, E-UMCU-11, E-GEOD-13601, GDS1062, GDS1584, GDS2520, GSE9349 and GSE9844), and selected the top 2.5% genes upregulated in OSCC tissues compared with non-cancerous tissues. We then integrated this gene set with the 248 true LMr proteins identified in our analysis (Table S2). From this comparison, we identified 33 LMr proteins that were present in both datasets as possible candidate OSCC biomarkers for further verification (Fig. 3A and Table 1). To prioritize targets for further verification, we first selected targets whose protein expression and/or functional roles have not been well characterized in OSCC but excluded the chemokine family proteins as most of them have been extensively studied before in many cancers including OSCC. We tested more than 20 commercially available antibodies, ultimately obtaining antibodies with high specificity against the following eight targets: macrophage migration inhibitory factor (MIF), high mobility group protein HMGI-C (HMGA2), activated RNA polymerase II transcriptional coactivator p15 (SUB1), transcription elongation factor B polypeptide 1 (TCEB1), small nuclear ribonucleoprotein F (SNRPF), 10 kDa heat shock protein, mitochondrial (HSPE1), serum amyloid A protein (SAA1) and lymphocyte antigen 6D (LY6D). Using these antibodies, we first confirmed the presence of the corresponding proteins in the conditioned media of the four OSCC cell lines by immunoblotting. As shown in Figure S3, these eight targets could be clearly detected in OSCC cell-conditioned media. Next, we examined the applicability of these antibodies in immunohistochemical staining of a small set of OSCC tissue sections and found that three antibodies against MIF, HMGA2 and HSPE1, respectively, showed high-quality immunohistochemical staining pattern in this preliminary test (Figure S4). Finally, we selected MIF and HMGA2 for subsequent study using a large OSCC tissue sample set.

### Patient characteristics

For the current study, we enrolled 191 males and 24 females who had been diagnosed with OSCC at ages ranging from 21.9 to 85.0 years (mean, 51.1 ± 11.8 years). The associated subsites were: buccal mucosa (74 patients), gum (27), hard palate (5), lip (7), floor of the mouth (12), and tongue (90). Twenty-four, 55, 33, and 103 of the enrolled patients had stage I, II, III, and IV diseases, respectively.

### HMGA2 and MIF are overexpressed in tumor cells of OSCC specimens

**Figure 4:**
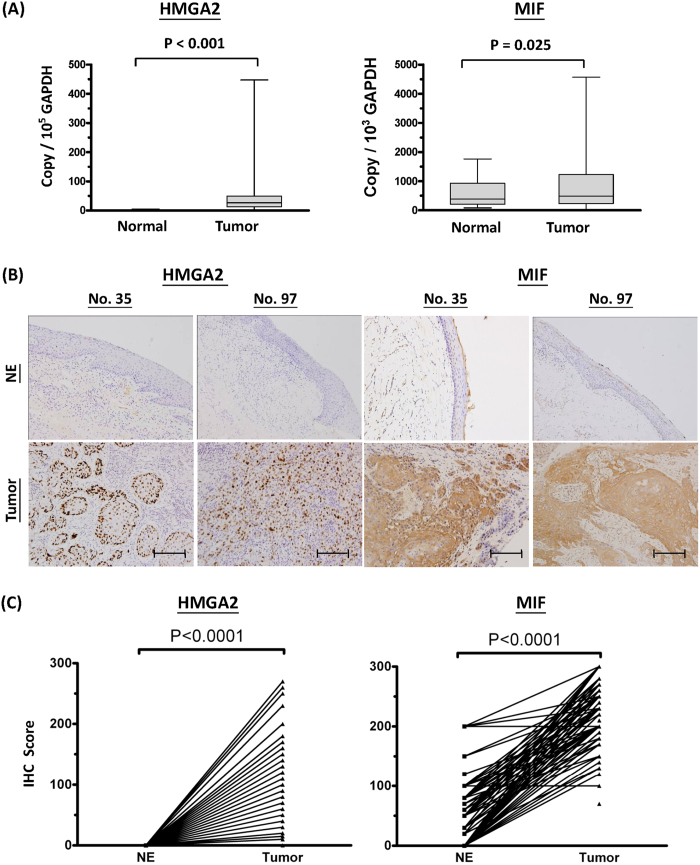
Overexpression of HMGA2 and MIF in OSCC tissues. (**A**) Box-plot analysis of HMGA2 and MIF mRNA transcript levels in the 40 paired pericancerous adjacent normal and tumor tissues as assessed by quantitative real-time PCR. The glyceraldehyde 3-phosphate dehydrogenase (GADPH) gene was used as an internal control for normalization. (**B**) Immunohistochemical staining of HMGA2 and MIF in pericancerous adjacent normal epithelia (NE) and tumor tissues from two representative cases (scale bar = 100 μm). The staining patterns (brown color) indicate that HMGA2 and MIF are localized in the nucleus and cytoplasm, respectively, of tumor cells. (**C**) Statistical analysis of the immunohistochemical scores of HMGA2 and MIF expression in 199 paired samples. Significantly higher expression levels of HMGA2 and MIF were observed in tumor cells compared to NE (60.9 ± 46.8 *vs*. 0, *P* < 0.0001 and 223.3 ± 42.8 *vs*. 55.9 ± 50.6, *P* < 0.0001, respectively). HMGA2 expression was not detected in normal oral epithelia.

Quantitative real-time PCR analysis of 40 paired OSCC tumor and adjacent normal tissue samples revealed that the transcript levels of *HMGA2* and *MIF* were significantly elevated in OSCC tumor specimens compared with adjacent normal tissues (48 ± 75 *vs.* 1 ± 1.5 copy/ 10^5^ GAPDH copy, *P* < 0.001 and 905 ± 965 *vs.* 562 ± 438 copy/ 10^3^ GAPDH copy, *P* = 0.025, respectively; Fig. 4A). Immunohistochemical staining of tissue sections revealed that the HMGA2 protein was exclusively expressed in the nucleus of tumor cells and was completely absent in the epithelia of the paired adjacent normal samples (Fig. 4B), whereas MIF was highly expressed in the cytoplasm of tumor cells, but the paired adjacent normal epithelium samples showed little or no expression of MIF (Fig. 4B). Statistical analysis of the immunohistochemical staining scores obtained from 199 paired samples revealed that the protein expression levels of HMGA2 and MIF were significantly higher in tumor cells compared to the non-tumor epithelia (60.9 ± 46.8 *vs*. 0, *P* < 0.001 and 223.3 ± 42.8 *vs*. 55.9 ± 50.6, *P* < 0.001, respectively; Fig. 4C). Notably, HMGA2 expression was not detected in normal oral epithelia. These findings indicate that MIF is more highly expressed in OSCC tumor cells compared to normal oral epithelia, and HMGA2 is specifically expressed in the nuclei of OSCC tumor cells.

### Association of HMGA2 and MIF expression with various clinicopathological manifestations

Next, we evaluated the relationships between increased levels of HMGA2 and MIF expression and various clinicopathological characteristics of our OSCC patients (Table 2). Positive HMGA2 expression was significantly associated with a higher pT status, a higher pN status, a higher overall pathological stage, poorer cell differentiation, positive perineural invasion, and greater tumor depth (*P* = 0.036, < 0.001, < 0.003, < 0.001, < 0.001, and < 0.001, respectively; Table 2). Higher MIF expression was significantly associated with a higher pT status, a higher pN status, a higher overall pathological stage, positive perineural invasion, and greater tumor depth (*P* < 0.001 for all; Table 2). In contrast, no significant association was observed between the overexpression of either proteins in OSCC tumors and patient age and gender.

### Association of HMGA2 and MIF overexpression with overall survival (OS), disease-free survival (DFS), and disease-specific survival (DSS)

**Figure 5:**
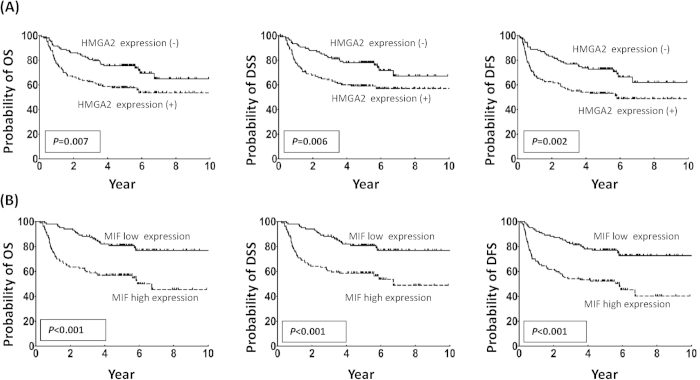
HMGA2 expression and high MIF expression are associated with a poorer prognosis for patient survival. (A) A Kaplan-Meier plot of overall survival indicates that the 5-year overall survival (OS), disease-specific survival (DSS), and disease-free survival (DFS) rates for patient subgroups stratified by the absence or presence of HMGA2 expression were 75.6% versus 57.7% (*P* = 0.007), 78% versus 59.1% (*P* = 0.006), and 72.7% versus 53.1% (*P* = 0.002), respectively. (**B**) A Kaplan-Meier plot of overall survival indicates that the 5-year OS, DSS, and DFS rates for patient subgroups stratified by low or high MIF expression were 80.8% versus 56.9% (*P* < 0.001), 80.8% versus 58.5% (*P* < 0.001), and 76.9% versus 52.2% (*P* < 0.001), respectively.

Based on the expression data obtained from our immunohistochemical staining experiments, patients were stratified into groups representing present versus absent nuclear staining for HMGA2 and high versus low MIF expression (using 160 out of 300 as the cut-off value). Survival analysis using Kaplan-Meier plots revealed that the 5-year OS rates for the stratified patients were 75.6% versus 57.7% and 80.8% versus 56.9%, respectively (*P* = 0.007 and < 0.001, respectively, by log-rank test); the 5-year DSS rates were 78.1% versus 59.1% and 80.8% versus 58.5%, respectively (*P* = 0.006 and < 0.001, respectively, by log-rank test); and the 5-year DFS rates were 72.7% versus 53.1% and 76.9% versus 52.2%, respectively (*P* = 0.002 and < 0.001, respectively) (Fig. 5). To further ascertain whether the overexpression of HMGA2 and/or MIF could be used as an independent predictor of patient survival, we performed a multivariate analysis using age, gender, overall stage, perineural invasion, and HGMA2/MIF overexpression as parameters in a Cox proportional regression model. Our results indicated that HMGA2 expression and MIF overexpression are both independent predictors of OS, DSS, and DFS (*P* = 0.028, 0.025, and 0.015, respectively, for HMGA2; *P* = 0.002, 0.006, and 0.002, respectively, for MIF)(data not shown).

### HMGA2 and MIF promote oral cancer cell migration and invasiveness *in vitro*

**Figure 6:**
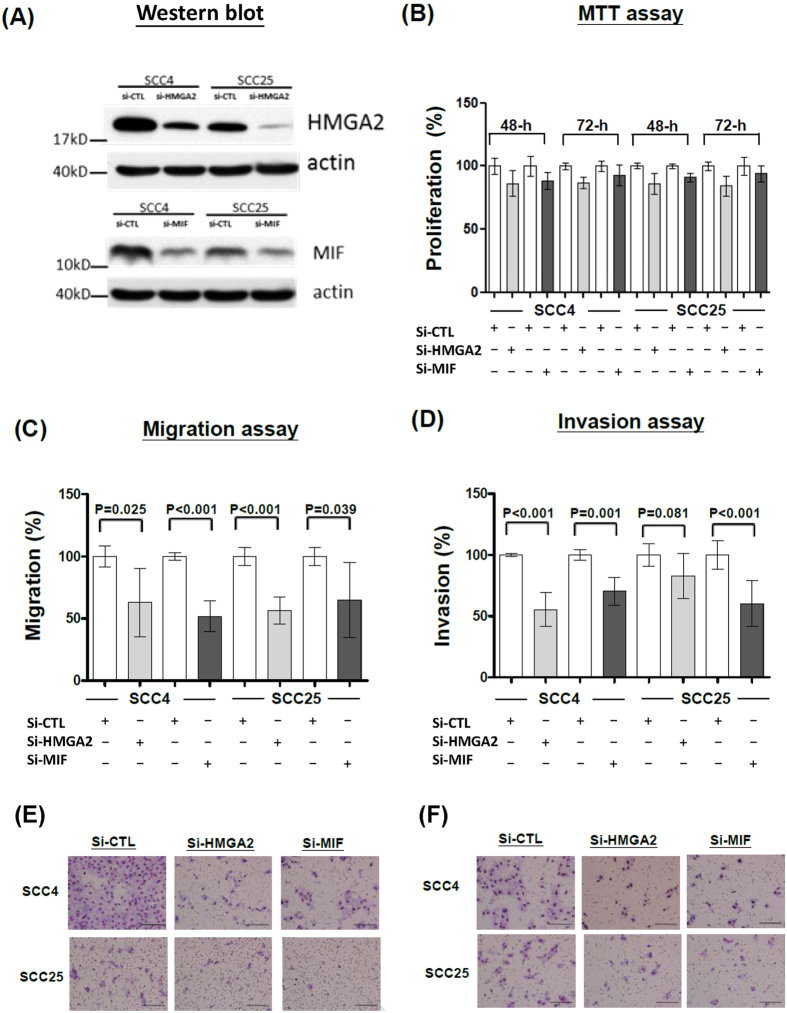
Attenuation of OSCC cell migration and invasiveness by *HGMA2*- and *MIF*-specific RNAi. (**A**) Western blot analysis of HGMA2 and MIF expression in lysates from SCC4 and SCC25 cells transfected with *HMGA2*-specific RNAi (si-HGMA2), *MIF*-specific RNAi (si-MIF), or the scrambled sequence control RNAi. (**B**) Cell proliferation assays for SCC4 and SCC25 cells transfected with si-HMGA2, si-MIF or the scrambled sequence control RNAi (si-CTL) for 48 and 72 h, respectively. (**C** and **D**) Cell migration and invasion assays for SCC4 and SCC25 cells transfected with si-HMGA2, si-MIF or si-CTL for 48 and 72 h, respectively. **(E** and **F)** Slides showing representative results of our cell migration and invasion assays.

The protein expression levels of endogenous HMGA2 and MIF and the effects of their siRNA-mediated silencing in two oral cancer cell lines (SCC4 and SCC25) were determined by Western blotting using anti-HMGA2 and anti-MIF antibodies. Significant decreases in the endogenous protein levels of HMGA2 and MIF were observed in cells transfected with individual siRNAs against HMGA2 and MIF, respectively, compared to those transfected with a control scrambled siRNA (Fig. 6A). Knockdown of HMGA2 or MIF had only marginal effects (~10%) on the proliferative ability of SCC4 and SCC25 cells (Fig. 6B), but significantly attenuated their cell migration (~40% reduction; Fig. 6C) and invasion (~35% reduction; Fig. 6D). These findings indicate that HMGA2 and MIF are both involved in regulating the migration and invasiveness of oral cancer cells.

## Discussion

Studies have shown that cancer cell secretome profiling is a viable strategy for identifying cancer-related biomarkers that are accessible in body fluids. We have used this strategy to discover some useful biomarkers based on the secretome profiling of cancer cell lines derived from OSCC tumors and nasopharyngeal carcinoma^15^,^18^,^20^,^21^,^22^. However, traditional protein separations on 8–14% gradient SDS gels usually fail to harvest the LMr proteins (<15 kDa) that are typically collected in the condition medium. Thus, such studies may miss some potentially important cancer-related LMr proteins. In the present study, we applied tricine–SDS-gel-assisted fractionation in conjunction with LC–MS/MS to systematically identify LMr proteins in the secretomes of five OSCC cell lines. To the best of our knowledge, very few previous studies have analyzed LMr proteins in cancer cell secretomes. We previously used a similar approach to analyze the LMr secretome derived from nasopharyngeal carcinoma cell lines, and successfully identified CCL5 as a potential plasma biomarker and therapeutic target for nasopharyngeal carcinoma^23^. In another study, Cao *et al.* sought to enrich and identify LMr proteins in the secretome of a human hepatocellular carcinoma cell line. Using a nanozeolite-assisted capture approach coupled with GeLC-MS/MS, the authors identified a total of 1474 unique proteins, 97 of which were <15 kDa^24^.

To identify the LMr proteins that were specifically overexpressed in OSCC tumor cells compared to normal epithelium, we used our previously described strategy^20^,^21^,^23^. We compared the 248 identified LMr proteins to those found in an OSCC tissue transcriptome database, and discovered the proteins that were present in both datasets as potential OSCC-specific LMr proteins. We therefore identified 33 candidate OSCC-related secreted LMr proteins, and further validated the overexpressions of two such proteins, HMGA2 and MIF, in OSCC tissues from a cohort of 215 OSCC patients. We have examined the presence of MIF and HMGA2 in the conditioned medium of OSCC cell lines by Western blot, and the results showed that both MIF and HMGA2 could be clearly detected in the conditioned media of all and two of four OSCC cell lines tested, respectively (Figure S3), indicating that these two proteins could be secreted/released from OSCC cells.

HMGA2 (high-motility group AT-hook 2), which is encoded by a gene located at chromosome 12q15, belongs to the non-histone chromosomal high mobility group (HMG) protein family, contains structural DNA-binding domains, and may act as a transcriptional regulator. HMGA2 is reportedly overexpressed in a variety of human neoplasms, including glioma, ovarian cancer, and colorectal cancer, and this overexpression has been associated with cancer cell migration, invasion, proliferation, and a poorer patient prognosis^25^,^26^,^27^. HMGA2 overexpression has also been correlated with E-cadherin loss and vimentin up-regulation during the epithelial-to-mesenchymal transition; these effects are activated via the TGFbeta signaling pathway and have been shown to induce the invasion and metastasis of human epithelial cancers^28^,^29^. Here, we report that HMGA2 is overexpressed in OSCC cells but undetectable in pericancerous normal epithelia (Fig. 4), strongly suggesting that HMGA2 is involved in the carcinogenesis of OSCC. This notion is further supported by our findings that positive HGMA2 staining in oral cancer cells is associated with many clinicopathological parameters (e.g., cervical metastasis), and the siRNA-mediated knockdown of HMGA2 attenuated in the migration and invasion capability of OSCC cells (Table 2 and Fig. 6). Finally, we found that HGMA2 overexpression appeared to be a strong prognosticator of oral cancer in our univariate and multivariate survival analyses. Together, these findings suggest that HMGA2 overexpression may be a useful clinical biomarker for OSCC.

The second validated candidate protein, MIF (macrophage migration inhibitory factor), is encoded by a gene located at chromosome 22q11.23. It is a lymphokine (a protein type that is rarely identified by the usual protein separation methods) that is involved in immunoregulation and inflammation. MIF is functionally unique among the cytokines; it acts upon multiple processes that are fundamental to tumorigenesis (e.g., tumor proliferation, evasion of apoptosis, angiogenesis and invasion) by activating the ERK-1/2 and AKT pathways and regulating JAB1, p53, SCF ubiquitin ligases, and HIF-1^30^,^31^. The significance of these pro-tumorigenic properties is reflected by the positive associations identified between MIF production and tumor aggressiveness/metastatic potential in the *in vitro* and *in vivo* models of some human tumors^31^,^32^,^33^,^34^. In OSCC, a recent study demonstrated that the salivary and serum levels of MIF decreased significantly after surgical resection in 50 OSCC patients, and the authors suggested that serological MIF levels could be considered as a marker of OSCC recurrence^35^. However, our previous study showed that MIF plasma levels did not significantly differ between OSCC patients and controls^36^. In the present study, we were unable to detect any significant difference in salivary MIF levels between OSCC patients and healthy controls using a commercially available ELISA kit (data not shown). However, our quantitative real-time PCR and immunohistochemistry experiments showed that MIF was overexpressed in OSCC tumors. We also found that higher MIF expression in oral cancer cells was associated with many clinicopathological manifestations related to more aggressive tumor properties (e.g., cervical metastasis, perineural invasion, and deeper tumor invasion depth), and that siRNA-mediated silencing of MIF *in vitro* attenuated the migration and invasion capability in OSCC cells (Table 2 and Fig. 6). Finally, higher MIF expression was associated with a poorer prognosis in our univariate and multivariate survival analyses. Together, these findings indicate that MIF expression may be a clinically relevant tissue marker of OSCC.

Although our data showed that both MIF and HMGA2 can be detected in the OSCC cell conditioned media, the exact mechanisms of how these two signal peptide-less proteins can be secreted/released by OSCC cells remain unclear at present. A previous study by Keller *et al.*^37^ provided important clue about the potential mechanism for secretion of signal peptide-less proteins in human keratinocytes. They reported that secretion of the signal peptide-less proteins proIL-1α, caspase-1, and fibroblast growth factor-2 depends on caspase-1 activity. Further secretome analysis using iTRAQ proteomics revealed caspase-1-mediated secretion of other signal peptide-less proteins with known or unknown extracellular functions, including MIF and HMGA2 (see Table 1 of Ref. 37). Additionally, several previous studies have reported the identification of MIF in exosomes derived from a variety of cell types, including B cells, bladder cancer cells and colorectal cancer cells^38^,^39^,^40^. Taken together, these observations suggest that the caspase-1-mediated, exosome-based secretion pathway may represent one of the potential mechanisms for secretion of MIF and HMGA2 from OSCC cells. This obviously represents an intriguing question that deserves further investigation.

## Conclusion

The findings of the present study collectively suggest that our approach provides a feasible strategy and a useful database for discovering novel OSCC cell-related LMr proteins and their related functional mechanisms. With regard to OSCC survival, both univariate and multivariate analyses showed that the overexpressions of HMGA2 and MIF were associated with a poorer prognosis, supporting the potential usefulness of these LMr proteins as prognostic biomarkers for OSCC tumors. Although we have successfully identified two novel proteins and pivotal pathways that may be associated with OSCC tumors, future work is warranted to examine additional candidate LMr proteins and their related mechanisms, in the hopes of achieving a more integrated understanding of OSCC carcinogenesis. Furthermore, to support the application of these experimental results to the clinical management of OSCC tumors, clinical studies should be performed on a prospective cohort of OSCC patients.

## Methods

### Patient characteristics and clinical specimens

Clinical samples for immunohistochemical analysis were obtained from a consecutive cohort of 215 OSCC patients diagnosed at the Chang Gung Memorial Hospital (Tao-Yuan, Taiwan) from August 2002 to December 2008. OSCC patients with unresectable disease, synchronous cancers, distant metastasis, or any previous history of malignancy were excluded. All patients provided informed consent prior to their participation, and this study was approved by the Institutional Review Board. All experiments were performed in accordance with the approved guidelines. According to the institution’s guidelines, each patient underwent a standard preoperative work-up that included a detailed medical history, a complete physical examination, computed tomography or magnetic resonance imaging scans of the head and neck, chest radiographs, a bone scan, and an abdominal ultrasound. Primary tumors were intraoperatively excised with adequate margins under frozen-section control. Classic radical or modified neck dissection (levels I–V) was performed in patients with clinically positive lymph node disease. Supraomohyoid neck dissection (levels I–III) was performed in clinically node-negative patients^41^. When necessary, surgical defects were immediately reconstructed by plastic surgeons using free or local flap techniques. The pathological and nodal stages of all tumors were established as described in the AJCC Cancer Staging Manual (2010). Post-operative radiotherapy was performed within 6 weeks following surgery on patients with pathologic T4 tumors and positive lymph nodes. Patients with pathologic evidence of multiple neck lymph node metastasis and/or extracapsular spread received concurrent adjuvant chemoradiotherapy (cisplatin plus a total radiation dose of 66 Gy given as 1.8-2 Gy per day, 5 days per week). After discharge, all patients had regular follow-up visits every 2 months for the first year, every 3 months for the second year, and every 6 months thereafter^14^,^42^.

### Cell culture and the harvest of secreted/shed proteins from conditioned media

The OEC-M1 oral epidermal carcinoma cell line was cultured in RPMI 1640 (Sigma-Aldrich); the SCC4 and SCC25 tongue squamous cell carcinoma cell lines were grown in DMEM/F12 (Invitrogen, Carlsbad, CA); the SAS tongue cancer cell line was maintained in DMEM supplemented with 10% heat-inactivated fetal bovine serum and 100 units/ml of penicillin/streptomycin (Invitrogen); and the OC3 human oral cancer cell line was established and cultured in DMEM and Keratinocyte-SFM (1:2 ratio) supplemented with 100 units/ml of penicillin/streptomycin. Cells were grown to approximately 80% confluence in 150-mm culture dishes (Corning Inc., Corning, NY), washed three times with 10 ml serum-free medium, and incubated at 37°C for 24 h in serum-free medium. Conditioned media were collected and centrifuged for 20 min at 1710 x g. The supernatants were concentrated and desalted with Amicon Ultra-15 tubes (molecular mass cutoff, 3,000 Da; Millipore, Billerica, MA), and then treated with a proteinase inhibitor cocktail (1 mM phenylmethylsulfonyl fluoride, 1 mM benzamidine, and 0.5 μg/ml leupeptin). The protein concentration of each supernatant was determined with a BCA protein assay reagent (Thermo Scientific, Rockford, IL, USA). The collected conditioned media were stored at−80°C until use.

### Tricine-sodium dodecyl sulfate-polyacrylamide gel electrophoresis (tricine-SDS-PAGE)

Tricine-SDS-PAGE was performed as previously described by Schagger^17^. Briefly, proteins were separated on large cast gels (dimensions: 0.15 × 14 × 14 cm) that consisted of an 8-cm separating gel (16% with 6 M Urea) overlaid with a 4-cm spacer gel (10%) followed by a stacking gel (4%). Equal amounts of protein (500 μg) were resolved at 4°C, and the gels were stained with Coomassie Brilliant Blue.

### In-gel tryptic digestion and mass spectrometric analysis

Selected Coomassie Brilliant Blue-stained protein bands were excised from the gel, destained three times (15 min each time) with 40% acetonitrile containing 25 mM ammonium bicarbonate, reduced by incubation at 60 °C for 30 min with 5 mM dithiothreitol, and then alkylated by incubation at room temperature in the dark for 30 min with 15 mM iodoacetamide. The proteins were in-gel digested at 37 °C for 16 h with freshly prepared trypsin solution (20 μg/ml of trypsin in 25 mM ammonium bicarbonate), and then extracted with 100% acetonitrile containing 1% formic acid. Finally, the extracted tryptic peptides were concentrated with a SpeedVac. Peptide samples were reconstituted with 0.1% formic acid, and then separated and analyzed on a nanoLC-LTQ-Orbitrap hybrid mass spectrometer (Thermo Scientific, San Jose, CA, USA), as described previously^18^. Intact peptides were detected in the Orbitrap at a resolution of 30,000. For internal calibration, we used the ion signal of (Si(CH_3_)2O)6H + at m/z 445.120025 as a lock mass. A data-dependent procedure that alternated between one MS scan and six MS/MS scans was applied for the six most abundant precursor ions identified in the MS survey. The m/z values selected for MS/MS were dynamically excluded for 180 s. Single microscans with maximum fill times of 1000 and 100 ms were used to acquire the MS and MS/MS spectra, respectively. Automatic gain control was used to prevent over-filling of the ion trap; 5 × 10^4^ ions were accumulated in the ion trap for generation of MS/MS spectra. For MS scans, the m/z scan range was 350–2000 Da.

### Database searches and bioinformatic analysis

The Mascot generic format (MGF) peak list files were created by processing the raw MS data files with the DTASuperCharge software (version 1.19). The peak lists were then searched against *Homo sapiens* entries in the Swiss-Prot database (released June 15, 2010; 20,306 entries) using MASCOT Daemon (version 2.2.2; Matrix Science, London, UK). The enzyme specificity parameter was set to “trypsin” and two missed cleavages were allowed. Carbamidomethylation of cysteines was set as a fixed modification, and oxidations of methionine, acetyl (protein N-term) and Gln-> pyro-Glu (N-term Q) were set as variable modifications. The MS/MS tolerance was set to 0.5 Da, and the mass tolerance for the monoisotopic peptide window was set to 10 ppm. The Scaffold software package (version 2.02.01; Proteome Software Inc., Portland, OR, USA) was used to combine all DAT files from the Mascot search and evaluate the MS/MS-based peptide and protein identifications. The threshold for protein identification was set to >95%, and we assumed a peptide identification probability >95% and more than two unique identified peptides.

We measured the false-positive rate of peptide identification by searching a random database in which every sequence entry from the “normal” database had been randomly shuffled. The number of hits from each search was categorized based on the score, and for each scoring interval, the false-positive rate was calculated as the number of random hits/(number of random hits + number of normal hits). In the present study, the false-positive rate for peptide sequence matches obtained using this strategy was estimated to be <0.1%.

The SignalP and SecretomeP programs were used to predict secretory signal peptides and non-signal peptide-mediated secretion, respectively, for the LMr proteins identified in conditioned media from our OSCC cell cultures. ^21, 22^ Transmembrane helices were predicted using the TMHMM program^43^.

### Meta-analysis

Nine oral tissue gene expression datasets (GDS1062, GDS1584, GDS2520, GSE9349, GSE9844, E-MEXP-44, E-TABM-302, E-UMCU-11 and E-GEOD-13601) were retrieved from the National Center for Biotechnology Information (NCBI), Gene Expression Omnibus (GEO) (http://www.ncbi.nlm.nih.gov/geo) or ArrayExpress (https://www.ebi.ac.uk/arrayexpress) and two-sample *t*-tests were used to identify genes whose expression levels differed significantly between OSCC and non-cancerous oral epithelial tissues (*P* < 0.05)^44^,^45^,^46^,^47^,^48^,^49^,^50^,^51^. We calculated the tumor/normal (T/N) ratios using the mean intensities of each gene probe in the healthy and cancerous groups, and ranked this ratio to obtain the top 2.5% genes upregulated in OSCC tissues compared to non-cancerous oral epithelial tissues. We then converted the microarray probe set IDs to Swiss-Prot IDs and matched them with the LMr proteins that we had newly identified in the OSCC cell secretomes, in order to identify candidate biomarkers for further verification.

### RNA extraction and quantitative real-time RT-PCR

Forty paired OSCC tumor and pericancerous normal tissues were individually homogenized in liquid nitrogen with a mortar and pestle, and total RNA was extracted with RNAzol B (Tel-Test, Friendswood, TX) according to the manufacturer’s protocol. The RNA was further purified using an RNeasy cleanup kit (Qiagen, Valencia, CA). First-strand cDNA was synthesized from 5 μg of total RNA and then mixed with commercially available primers (HMGA2 Hs00171569_m1, MIF Hs00236988_g1 and normalization control GAPDH, Hs99999905_m1; Assay-on-Demand, Applied Biosystems, Foster City, CA), RNase-free water, and TaqMan Universal PCR Master Mix (Applied Biosystems). Quantitative real-time RT-PCR was performed on a 7900 HT Sequence Detection System, and the results were analyzed using the SDS software, version 2 (both from Applied Biosystems). All experiments were performed in duplicate, and the mean fold-change was calculated for each sample.

### Immunohistochemical staining

For immunohistochemistry, formalin-fixed and paraffin-embedded tissues were cut into 4-μm sections, deparaffinized, rehydrated, and prepared for antigen retrieval. Slides of consecutive sections were incubated with either rabbit monoclonal anti-HMGA2 (D1A7; diluted 1:30, #8179; Cell Signaling, Danvers, MA) or rabbit polyclonal anti-MIF (FL-115; diluted 1:800, sc-20121; Santa Cruz Biotech, Dallas, TX). The slides were then washed three times with phosphate buffered saline (PBS), incubated at room temperature for 10 min with horseradish peroxidase (HRP) polymer antibody (Invitrogen, Carlsbad, CA), and developed by the addition of 3,3′-diaminobenzidine tetrahydrochloride (DAB) reagent (Dako, Glostrup, Denmark) as the chromogen and hematoxylin as the counterstain. Images of stained slides were obtained using a ScanScope CT automated slide-scanning system (Aperio Technologies, Vista, CA). The expression level of HMGA2 or MIF was scored using a combined scoring method that accounted for both the staining intensity and the percentage of stained cells, as previously described^14^,^20^,^52^,^53^,^54^. Strong, moderate, weak, and negative staining intensities were scored as 3, 2, 1, and 0, respectively. For each intensity score, cells staining at that specific level were visually estimated and calculated as a percentage. The combined score was calculated as the sum of the percentage of stained cells multiplied by the intensity scores. All specimens were independently evaluated by our pathologist (Liang Y), who had no prior knowledge of the clinical origin of any specimen.

### Western blot analysis

Proteins were extracted from cultured cells with RIPA buffer [50 mM Tris, pH 8, 0.0150 mM NaCl, 2 mM EDTA, 1% Triton X-100, 0.1% SDS, 0.2% Na-deoxylate 1x protease cocktail (Sigma-Aldrich, St. Louis, MO)], and the protein concentration was determined using a BCA protein assay kit (Thermo Scientific, Rockford, IL, USA). Samples were separated on 12% SDS gels, transferred to PVDF membranes (GE Healthcare Life Sciences, Buckinghamshire, UK), and probed using the rabbit monoclonal anti-HMGA2 antibody (Cell Signaling, Danvers, MA), the rabbit polyclonal MIF antibody (Santa Cruz Biotech, Dallas, TX), or a mouse monoclonal beta-actin antibody (MAB1501; Chemicon, Billerica, MA). The actin signal was used as the loading control.

### Cell culture, cell function assays (proliferation, migration, and invasion) and gene silencing via RNA interference

SMARTpool small interfering RNAs (siRNAs) were purchased from Thermo Scientific Dharmacon (Lafayette, CO). RNAi specifically targeting human *HMGA2* (No. L-013495-00-0005, Dharmacon), *MIF* (No. L-011335-00-0005, Dharmacon), and a scrambled control RNAi (No. D-001810-10-05, Dharmacon) were purchased from Thermo Fisher Scientific (Rockford, IL). RNAi (at a final concentration 400 nM) was incubated for 20 min at room temperature with Lipofectamine RNAiMAX (Invitrogen, Carlsbad, CA) and Opti-MEM medium (Invitrogen, Carlsbad, CA) without serum, incubated for 20 min at room temperature, and then added to SCC4 or SCC25 cells seeded to six-well plates (1 × 10^5^ cells per well). After incubation for 6 h at 37 °C, fresh DMEM/F12 medium containing 10% FBS was added to each well. After 48 h, the transfected cells were harvested for analysis of cell functions. Cell proliferation was determined by the 3-(4,5-dimethylthiazol-2-yl)-2,5-diphenyltetrazolium bromide (MTT) assay, and cell migration and invasion were examined in a Boyden chamber, as described in our previous report^53^.

### Statistical analysis

All statistical data are expressed as means ± SD. The Wilcoxon signed ranks test was used to compare the relative signal intensities (immunohistochemical staining scores) of paired tumor and pericancerous normal epithelium samples. Cell proliferation, migration, and invasion data were compared using the unpaired Student’s *t*-test. The associations of various clinicopathological parameters with the immunohistochemical scores for HMGA2 and MIF were evaluated using the Wilcoxon test. All statistical analyses were performed using the SAS software (version 9.1; SAS Institute Inc., Cary, NC). All patients received follow-up evaluations at our outpatient clinic until August 2010 or death. The survival time and various time intervals were calculated from the date of operation. Survival analyses were plotted using the Kaplan-Meier method, and differences were evaluated with the log-rank test. Univariate and multivariate regression analyses were performed under the Cox proportional hazard model, and were employed to define specific risk factors for survival status. All *P* values were two-sided, and statistical significance was accepted at *P* < 0.05.

## Additional Information

**How to cite this article**: Chang, K.-P. *et al.* Low-molecular-mass secretome profiling identifies HMGA2 and MIF as prognostic biomarkers for oral cavity squamous cell carcinoma. *Sci. Rep.* **5**, 11689; doi: 10.1038/srep11689 (2015).

## Supplementary Material

Supplementary Figures

Supplementary Table S1, S2, S3, S4, S5, S6

## Figures and Tables

**Table 1:** List of 33 potential OSCC biomarkers derived from LMr secretome.

Protein (Gene symbol)	MW(Da)	Detectable in LMr secretome					Upregulated in microarray dataset									Secretory pathway predicition		
		OC3	OECM1	SAS	SCC4	SCC25	E-MEXP-44	E-TABM-302	E-UMCU-11	GDS1062	GDS1584	GDS2520	E-GEOD-13601	GSE9349	GSE9844	SingnalP	SecrectomeP*	TMHMM
Beta-defensin 2 (DEFB4)	7038		+										+		+	Y	Y	Y
Ubiquitin (RPS27A)	8565	+	+		+	+	+								+	N	Y	N
Cyclin-dependent kinases regulatory subunit 1 (CKS1B)	9660	+	+		+	+	+					+			+	N	N	N
Small nuclear ribonucleoprotein F (SNRPF)	9725	+	+	+	+		+									N	N	N
Cyclin-dependent kinases regulatory subunit 2 (CKS2)	9860	+			+	+	+						+	+		N	N	N
C-X-C motif chemokine 11 (CXCL11)	10365	+	+			+		+			+		+		+	Y	N	Y
C-C motif chemokine 20 (CCL20)	10762	+	+	+		+							+		+	Y	Y	N
C-X-C motif chemokine 10 (CXCL10)	10882	+	+		+	+					+		+		+	Y	Y	Y
Growth-regulated alpha protein (CXCL1)	11302	+	+	+	+	+					+		+		+	Y	Y	N
C-X-C motif chemokine 2 (CXCL2)	11389	+	+	+		+									+	Y	Y	N
High mobility group protein HMGI-C (HMGA2)	11832	+			+	+					+				+	N	Y	N
Interferon-induced transmembrane protein 1 (IFITM1)	13965	+	+	+	+	+					+					N	Y	Y
Histone H2A type 2-A (HIST2H2AA3)	14096	+	+	+	+	+						+	+		+	N	N	N
Galectin-1 (LGALS1)	14716	+	+	+	+	+					+	+				N	N	N
Thymosin beta-10 (TMSB10)	5026	+	+	+	+	+							+			N	Y	N
Apolipoprotein C-I (APOC1)	9332	+	+	+	+	+					+					Y	Y	N
C-C motif chemokine 5 (CCL5)	9990	+	+		+	+					+		+			Y	Y	Y
BolA-like protein 2 (BOLA2)	10116	+	+	+	+	+	+						+			N	Y	N
60S ribosomal protein L37a (RPL37A)	10275	+			+	+				+						N	Y	N
Protein S100-P (S100P)	10400	+	+	+	+	+							+			N	Y	N
10 kDa heat shock protein, mitochondrial (HSPE1)	10932	+	+	+	+	+						+				N	Y	N
Protein S100-A2 (S100A2)	11117	+	+	+	+	+							+			N	N	N
Apolipoprotein A-II (APOA2)	11175	+								+						Y	Y	N
C-X-C motif chemokine 3 (CXCL3)	11343	+	+	+	+	+									+	Y	Y	N
C-X-C motif chemokine 6 (CXCL6)	11898				+										+	Y	Y	N
Transcription elongation factor B polypeptide 1 (TCEB1)	12473	+	+	+	+	+	+									N	N	N
Macrophage migration inhibitory factor (MIF)	12477	+	+	+	+	+							+			N	Y	N
Lymphocyte antigen 6D (LY6D)	13286	+			+	+							+			Y	N	Y
Histone H2A.V (H2AFV)	13509	+	+	+	+	+									+	N	Y	N
Serum amyloid A protein (SAA1)	13533	+			+	+		+			+				+	Y	Y	N
Histone H2B type 1-C/E/F/G/I (HIST1H2BC)	13893	+	+	+	+	+	+					+				N	N	N
Activated RNA polymerase II transcriptional coactivator p15 (SUB1)	14396	+	+	+	+	+							+			N	Y	N
Fatty acid-binding protein, adipocyte (FABP4)	14719					+					+					N	Y	N

^*^Y, NN-score ≥0.5; N, NN-score <0.5.

**Table 2:** Association of HMGA2 and MIF expression levels (immunohistochemical scores of three proteins increased in the OSCC tumors) with clinicopathological characteristics in 215 untreated OSCC patients.

No.		HMGA2		MIF	
		Immunohistochemical score*	p-value	Immunohistochemical score*	p-value
**Gender**					
Female	24	43 ± 51.301 (180, 0)	0.800	152 ± 44.026 (220, 50)	0.821
Male	191	47 ± 62.124 (270, 0)		153 ± 44.926 (230, 40)	
**Age**†					
<49.8	106	44 ± 56.491 (230, 12.5)	0.848	155 ± 41.605 (220, 50)	0.899
>49.8	109	49 ± 65.104 (270, 10)		152 ± 47.758 (230, 40)	
**pT Status**					
1–2	110	36 ± 53.253 (250, 10)	0.036	139 ± 48.589 (220, 40)	<0.001
3–4	105	58 ± 66.483 (270, 40)		169 ± 33.743 (230, 90)	
**pN Status**					
(–)	137	34 ± 53.053 (230, 10)	<0.001	145 ± 45.294 (220, 50)	<0.001
( + )	78	67 ± 68.126 (270, 50)		168 ± 39.750 (230, 40)	
**Overall Pathological Stage**					
I-II	79	29 ± 45.033 (170, 10)	0.003	133 ± 48.298 (220, 50)	<0.001
III-IV	136	57 ± 66.520 (270, 35)		165 ± 37.701 (230, 40)	
**Cell differentiation††**					
W-D + M-D	188	40 ± 54.131 (250, 10)	<0.001	152 ± 44.667 (230, 40)	0.078
P-D	26	98 ± 81.471 (270, 100)		168 ± 41.668 (220, 50)	
**Perineural Invsion**					
No	147	37 ± 56.375 (260, 10)	<0.001	144 ± 46.012 (220, 40)	<0.001
Yes	68	67 ± 65.548 (270, 50)		173 ± 34.264 (230, 50)	
**Tumor depth**†,††					
<=8	107	35 ± 56.832 (250, 0)	<0.001	139 ± 46.230 (220, 50)	<0.001
>8	107	58 ± 63.078 (270, 40)		169 ± 37.274 (230, 40)	

W-D: well-differentiated, M-D: moderatively-differentiated, and P-D: poorly-differentiated, squamous cell carcinoma.

^*^Mean ± SD, median (maximum, minimum).

^†^Median,

^††^data absent in one patient.

## References

[b1] Siegel R., Naishadham D. & Jemal A. Cancer statistics, 2013. CA Cancer J. Clin. 63, 11–30, 10.3322/caac.21166 (2013). 23335087

[b2] Jemal A. *et al.* Global cancer statistics. CA Cancer J. Clin. 61, 69–90, 10.3322/caac.20107 (2011). 21296855

[b3] Blot W. J. *et al.* Smoking and drinking in relation to oral and pharyngeal cancer. Cancer Res. 48, 3282–3287 (1988). 3365707

[b4] Hashibe M. *et al.* Interaction between tobacco and alcohol use and the risk of head and neck cancer: pooled analysis in the International Head and Neck Cancer Epidemiology Consortium. Cancer Epidemiol. Biomarkers Prev. 18, 541–550, 10.1158/1055-9965.EPI-08-0347 (2009). 19190158 PMC3051410

[b5] D’Souza G., Agrawal Y., Halpern J., Bodison S. & Gillison M. L. Oral sexual behaviors associated with prevalent oral human papillomavirus infection. J. Infect. Dis. 199, 1263–1269, 10.1086/597755 (2009). 19320589 PMC4703086

[b6] Ho P. S., Ko Y. C., Yang Y. H., Shieh T. Y. & Tsai C. C. The incidence of oropharyngeal cancer in Taiwan: an endemic betel quid chewing area. J. Oral Pathol. Med. 31, 213–219 (2002). 12076324 10.1034/j.1600-0714.2002.310404.x

[b7] Wen C. P. *et al.* Cancer risks from betel quid chewing beyond oral cancer: a multiple-site carcinogen when acting with smoking. Cancer Causes Control 21, 1427–1435, 10.1007/s10552-010-9570-1 (2010). 20458529

[b8] Funk G. F. *et al.* Presentation, treatment, and outcome of oral cavity cancer: a National Cancer Data Base report. Head Neck 24, 165–180 (2002). 11891947 10.1002/hed.10004

[b9] Diaz E. M. Jr., Holsinger F. C., Zuniga E. R., Roberts D. B. & Sorensen D. M. Squamous cell carcinoma of the buccal mucosa: one institution’s experience with 119 previously untreated patients. Head Neck 25, 267–273, 10.1002/hed.10221 (2003). 12658730

[b10] Muir C. & Weiland L. Upper aerodigestive tract cancers. Cancer 75, 147–153 (1995). 8000993 10.1002/1097-0142(19950101)75:1+<147::aid-cncr2820751304>3.0.co;2-u

[b11] Wong Y. K. *et al.* Socio-demographic factors in the prognosis of oral cancer patients. Oral Oncol. 42, 893–906, 10.1016/j.oraloncology.2005.12.007 (2006). 16730220

[b12] Scully C. & Bagan J. V. Recent advances in Oral Oncology. Oral Oncol. 43, 107–115, 10.1016/j.oraloncology.2006.12.007 (2007). 17275742

[b13] Zhen W. *et al.* The National Cancer Data Base report on squamous cell carcinoma of the base of tongue. Head Neck 26, 660–674, 10.1002/hed.20064 (2004). 15287033

[b14] Chang K. P. *et al.* Identification of PRDX4 and P4HA2 as metastasis-associated proteins in oral cavity squamous cell carcinoma by comparative tissue proteomics of microdissected specimens using iTRAQ technology. J. Proteome Res. 10, 4935–4947, 10.1021/pr200311p (2011). 21859152

[b15] Weng L. P. *et al.* Secretome-based identification of Mac-2 binding protein as a potential oral cancer marker involved in cell growth and motility. J. Proteome Res. 7, 3765–3775, 10.1021/pr800042n (2008). 18646789

[b16] Chi L. M. *et al.* Enhanced interferon signaling pathway in oral cancer revealed by quantitative proteome analysis of microdissected specimens using 16O/18O labeling and integrated two-dimensional LC-ESI-MALDI tandem MS. Mol. Cell. Proteomics 8, 1453–1474, M800460-MCP200/mcp.M800460-MCP200 (2009). 19297561 10.1074/mcp.M800460-MCP200 PMC2709179

[b17] Schagger H. Tricine-SDS-PAGE. Nat. Protoc. 1, 16–22, 10.1038/nprot.2006.4 (2006). 17406207

[b18] Wu C. C. *et al.* Candidate serological biomarkers for cancer identified from the secretomes of 23 cancer cell lines and the human protein atlas. Mol. Cell. Proteomics 9, 1100–1117, 10.1074/mcp.M900398-MCP200 (2010). 20124221 PMC2877973

[b19] Conesa A. *et al.* Blast2GO: a universal tool for annotation, visualization and analysis in functional genomics research. Bioinformatics 21, 3674–3676, 10.1093/bioinformatics/bti610 (2005). 16081474

[b20] Chang K. P. *et al.* Identification of candidate nasopharyngeal carcinoma serum biomarkers by cancer cell secretome and tissue transcriptome analysis: Potential usage of cystatin A for predicting nodal stage and poor prognosis. Proteomics , 10.1002/pmic.200900620 (2010). 20461718

[b21] Yu C. J. *et al.* Identification of guanylate-binding protein 1 as a potential oral cancer marker involved in cell invasion using omics-based analysis. J. Proteome Res. 10, 3778–3788, 10.1021/pr2004133 (2011). 21714544

[b22] Wu C. C. *et al.* Cancer cell-secreted proteomes as a basis for searching potential tumor markers: nasopharyngeal carcinoma as a model. Proteomics 5, 3173–3182, 10.1002/pmic.200401133 (2005). 16035111

[b23] Lin S. J. *et al.* Low-molecular-mass secretome profiling identifies C-C motif chemokine 5 as a potential plasma biomarker and therapeutic target for nasopharyngeal carcinoma. J. Proteomics 94, 186–201, 10.1016/j.jprot.2013.09.013 (2013). 24080422

[b24] Cao J. *et al.* Nanozeolite-driven approach for enrichment of secretory proteins in human hepatocellular carcinoma cells. Proteomics 9, 4881–4888, 10.1002/pmic.200800877 (2009). 19743415

[b25] Xi Y. N., Xin X. Y. & Ye H. M. Effects of HMGA2 on malignant degree, invasion, metastasis, proliferation and cellular morphology of ovarian cancer cells. Asian Pac. J. Trop. Med. 7, 289–292, 10.1016/S1995-7645(14)60040-7 (2014). 24507678

[b26] Liu B. *et al.* Expression of high-mobility group AT-hook protein 2 and its prognostic significance in malignant gliomas. Hum. Pathol. 45, 1752–1758, 10.1016/j.humpath.2014.02.028 (2014). 24935062

[b27] Wang X. *et al.* Overexpression of HMGA2 promotes metastasis and impacts survival of colorectal cancers. Clin. Cancer Res. 17, 2570–2580, 10.1158/1078-0432.CCR-10-2542 (2011). 21252160 PMC3079060

[b28] Morishita A. *et al.* HMGA2 is a driver of tumor metastasis. Cancer Res. 73, 4289–4299, 10.1158/0008-5472.CAN-12-3848 (2013). 23722545 PMC3715567

[b29] Ding X. *et al.* Expression of HMGA2 in bladder cancer and its association with epithelial-to-mesenchymal transition. Cell Prolif. 47, 146–151, 10.1111/cpr.12096 (2014). 24571540 PMC6496012

[b30] Bifulco C., McDaniel K., Leng L. & Bucala R. Tumor growth-promoting properties of macrophage migration inhibitory factor. Curr. Pharm. Des. 14, 3790–3801 (2008). 19128232 10.2174/138161208786898608

[b31] Oliveira C. S. *et al.* Macrophage migration inhibitory factor engages PI3K/Akt signalling and is a prognostic factor in metastatic melanoma. BMC Cancer 14, 630, 10.1186/1471-2407-14-630 (2014). 25168062 PMC4155090

[b32] Huang X. H. *et al.* Small interfering RNA (siRNA)-mediated knockdown of macrophage migration inhibitory factor (MIF) suppressed cyclin D1 expression and hepatocellular carcinoma cell proliferation. Oncotarget 5, 5570–5580 (2014). 25015194 10.18632/oncotarget.2141 PMC4170598

[b33] Morris K. T., Nofchissey R. A., Pinchuk I. V. & Beswick E. J. Chronic macrophage migration inhibitory factor exposure induces mesenchymal epithelial transition and promotes gastric and colon cancers. PLoS One 9, e98656, 10.1371/journal.pone.0098656 (2014). 24887129 PMC4041794

[b34] Choudhary S. *et al.* Macrophage migratory inhibitory factor promotes bladder cancer progression via increasing proliferation and angiogenesis. Carcinogenesis 34, 2891–2899, 10.1093/carcin/bgt239 (2013). 23825153 PMC3845890

[b35] DE Souza M. B., Curioni O. A., Kanda J. L. & DE Carvalho M. B. Serum and salivary macrophage migration inhibitory factor in patients with oral squamous cell carcinoma. Oncol. Lett. 8, 2267–2275, 10.3892/ol.2014.2513 (2014). 25289107 PMC4186499

[b36] Chang K. P. *et al.* Prognostic cytokine markers in peripheral blood for oral cavity squamous cell carcinoma identified by multiplexed immunobead-based profiling. Clin. Chim. Acta 412, 980–987, S0009-8981(11)00071-4/j.cca.2011.02.002 (2011). 21315702 10.1016/j.cca.2011.02.002

[b37] Keller M., Rüegg A., Werner S. & Beer H. D. Active caspase-1 is a regulator of unconventional protein secretion. Cell 132, 818–831. 10.1016/j.cell.2007.12.040 (2008). 18329368

[b38] Buschow S. I. *et al.* MHC class II-associated proteins in B-cell exosomes and potential functional implications for exosome biogenesis. Immunol. Cell Biol. 88, 851–856. 10.1038/icb.2010.64 (2010). 20458337

[b39] Welton J. L. *et al.* Proteomics analysis of bladder cancer exosomes. Mol. Cell. Proteomics 9, 1324–1338 (2010). 20224111 10.1074/mcp.M000063-MCP201 PMC2877990

[b40] Mathivanan S. *et al.* Proteomics analysis of A33 immunoaffinity-purified exosomes released from the human colon tumor cell line LIM1215 reveals a tissue-specific protein signature. Mol. Cell. Proteomics 9, 197–208 (2010). 19837982 10.1074/mcp.M900152-MCP200 PMC2830834

[b41] Liao C. T. *et al.* Surgical outcome of T4a and resected T4b oral cavity cancer. Cancer 107, 337–344, 10.1002/cncr.21984 (2006). 16770782

[b42] Fang K. H. *et al.* Histological differentiation of primary oral squamous cell carcinomas in an area of betel quid chewing prevalence. Otolaryngol. Head Neck Surg. 141, 743–749, S0194-5998(09)01507-1/j.otohns.2009.09.012 (2009). 19932848 10.1016/j.otohns.2009.09.012

[b43] Moller S., Croning M. D. & Apweiler R. Evaluation of methods for the prediction of membrane spanning regions. Bioinformatics 17, 646–653 (2001). 11448883 10.1093/bioinformatics/17.7.646

[b44] Rustici G. *et al.* ArrayExpress update--trends in database growth and links to data analysis tools. Nucleic Acids Res. 41, D987–990, 10.1093/nar/gks1174 (2013). 23193272 PMC3531147

[b45] Rickman D. S. *et al.* Prediction of future metastasis and molecular characterization of head and neck squamous-cell carcinoma based on transcriptome and genome analysis by microarrays. Oncogene 27, 6607–6622, 10.1038/onc.2008.251 (2008). 18679425

[b46] Estilo C. L. *et al.* Oral tongue cancer gene expression profiling: Identification of novel potential prognosticators by oligonucleotide microarray analysis. BMC Cancer 9, 11, 10.1186/1471-2407-9-11 (2009). PMC2649155 19138406

[b47] O’Donnell R. K. *et al.* Gene expression signature predicts lymphatic metastasis in squamous cell carcinoma of the oral cavity. Oncogene 24, 1244–1251, 10.1038/sj.onc.1208285 (2005). 15558013

[b48] Toruner G. A. *et al.* Association between gene expression profile and tumor invasion in oral squamous cell carcinoma. Cancer Genet. Cytogenet. 154, 27–35, 10.1016/j.cancergencyto.2004.01.026 (2004). 15381369

[b49] Kuriakose M. A. *et al.* Selection and validation of differentially expressed genes in head and neck cancer. Cell. Mol. Life Sci. 61, 1372–1383, 10.1007/s00018-004-4069-0 (2004). 15170515 PMC11138891

[b50] Hensen E. F. *et al.* Gene-expression of metastasized versus non-metastasized primary head and neck squamous cell carcinomas: a pathway-based analysis. BMC Cancer 8, 168, 1471-2407-8-168/1471-2407-8-168 (2008). 18544165 10.1186/1471-2407-8-168 PMC2438367

[b51] Ye H. *et al.* Transcriptomic dissection of tongue squamous cell carcinoma. BMC Genomics 9, 69, 1471-2164-9-69/1471-2164-9-69 (2008). 18254958 10.1186/1471-2164-9-69 PMC2262071

[b52] Chang K. P. *et al.* Macrophage inflammatory protein-3alpha is a novel serum marker for nasopharyngeal carcinoma detection and prediction of treatment outcomes. Clin. Cancer Res. 14, 6979–6987, 14/21/6979/1078-0432.CCR-08-0090 (2008). 18980993 10.1158/1078-0432.CCR-08-0090

[b53] Chang K. P. *et al.* Overexpression of activin A in oral squamous cell carcinoma: association with poor prognosis and tumor progression. Ann. Surg. Oncol. 17, 1945–1956, 10.1245/s10434-010-0926-2 (2010). 20309641

[b54] Chang K. P. *et al.* Overexpression of caldesmon is associated with lymph node metastasis and poorer prognosis in patients with oral cavity squamous cell carcinoma. Cancer 119, 4003–4011, 10.1002/cncr.28300 (2013). 23963810

