# Supplementary material for: Low-molecular-mass secretome profiling identifies HMGA2 and MIF as prognostic biomarkers for oral cavity squamous cell carcinoma
Source: Sci Rep. 2015 Jul 3;5:11689. doi: 10.1038/srep11689 (PMC4650660; doi:10.1038/srep11689)
Supplement: Supplementary Figures [file srep11689-s1.pdf]

Supplementary information

Low-molecular-mass secretome profiling identifies HMGA2 and MIF as prognostic biomarkers for oral cavity squamous cell carcinoma

Kai-Ping Chang[1, 3], Shih-Jie Lin[2], Shiau-Chin Liu[1], Jui-Shan Yi[1, 3], Kun-Yi Chien[2, 3], Lang-Ming Chi [3, 4], Huang-Kai Kao[5], Ying Liang[3], Yu-Tsun Lin[2], Yu-Sun Chang[2, 3] & Jau-Song Yu[2, 3, 6, *]

[1]Departments of Otolaryngology-Head & Neck Surgery, Chang Gung Memorial Hospital, Tao-Yuan, Taiwan; [2]Graduate Institute of Biomedical Sciences, College of Medicine, Chang Gung University, Tao-Yuan, Taiwan; [3]Molecular Medicine Research Center, Chang Gung University, Tao-Yuan, Taiwan; [4]Department of Medical Research, Chang Gung Memorial Hospital, Tao-Yuan, Taiwan; [5]Department of Plastic & Reconstructive Surgery, Chang Gung Memorial Hospital, Tao-Yuan, Taiwan; [6]Department of Cell and Molecular Biology, College of Medicine, Chang Gung University, Tao-Yuan, Taiwan.

Supplementary Figure 1

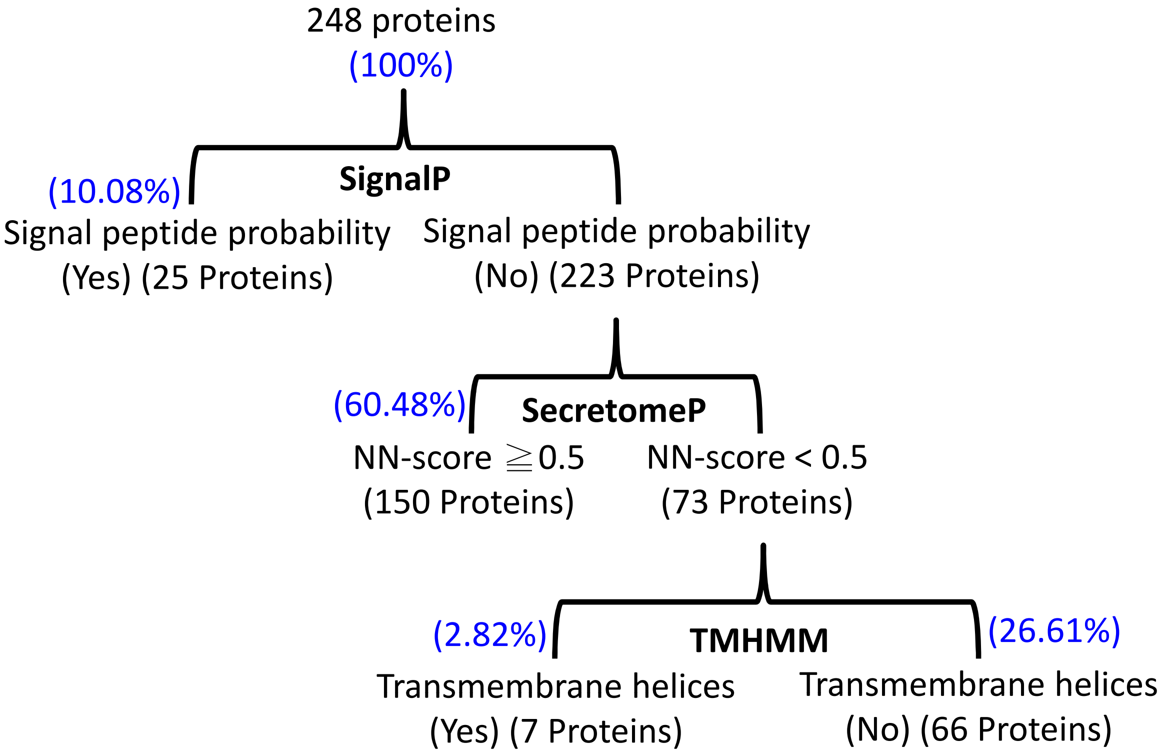

Supplementary Figure 1. Predicting the secretion pathways for the 248 identified LMr proteins. The SignalP software was used for secretory signal peptide prediction. All proteins lacking an apparent secretory signal peptide were analyzed with the SecretomeP program for the prediction of non-signal-peptide-triggered secretion. Finally, the remaining proteins were analyzed by TMHMM for prediction of transmembrane helices.

Supplementary Figure 2

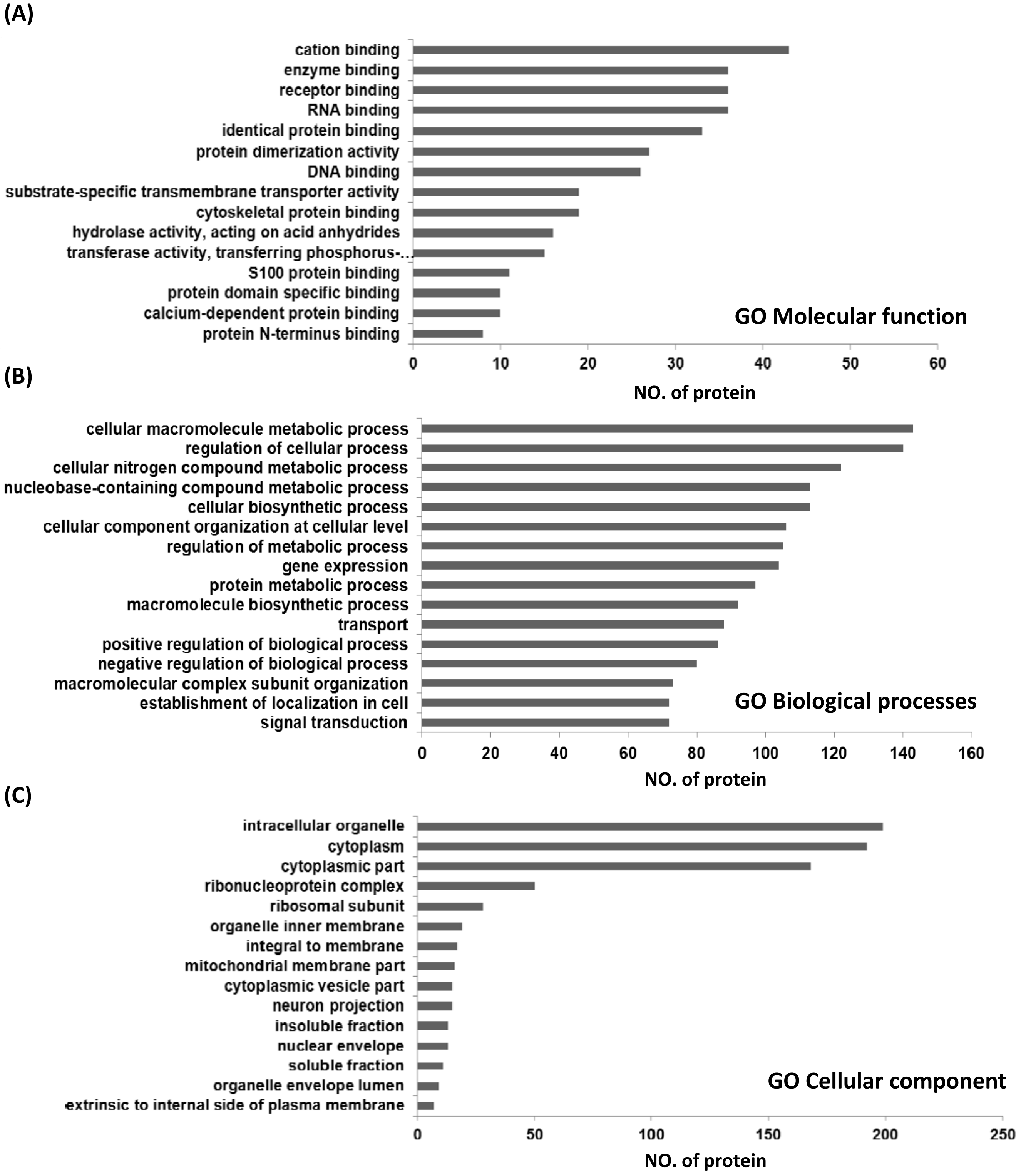

Supplementary Figure 2. Functional classification of the secreted LMr proteins identified in the conditioned media of five OSCC cell lines. The ProteinCenter software was used to classify the identified LMr proteins according to: (A) GO molecular function, (B) GO biological processes and (C) GO cellular components (as noted for at least one annotation term).

Supplementary Figure 3

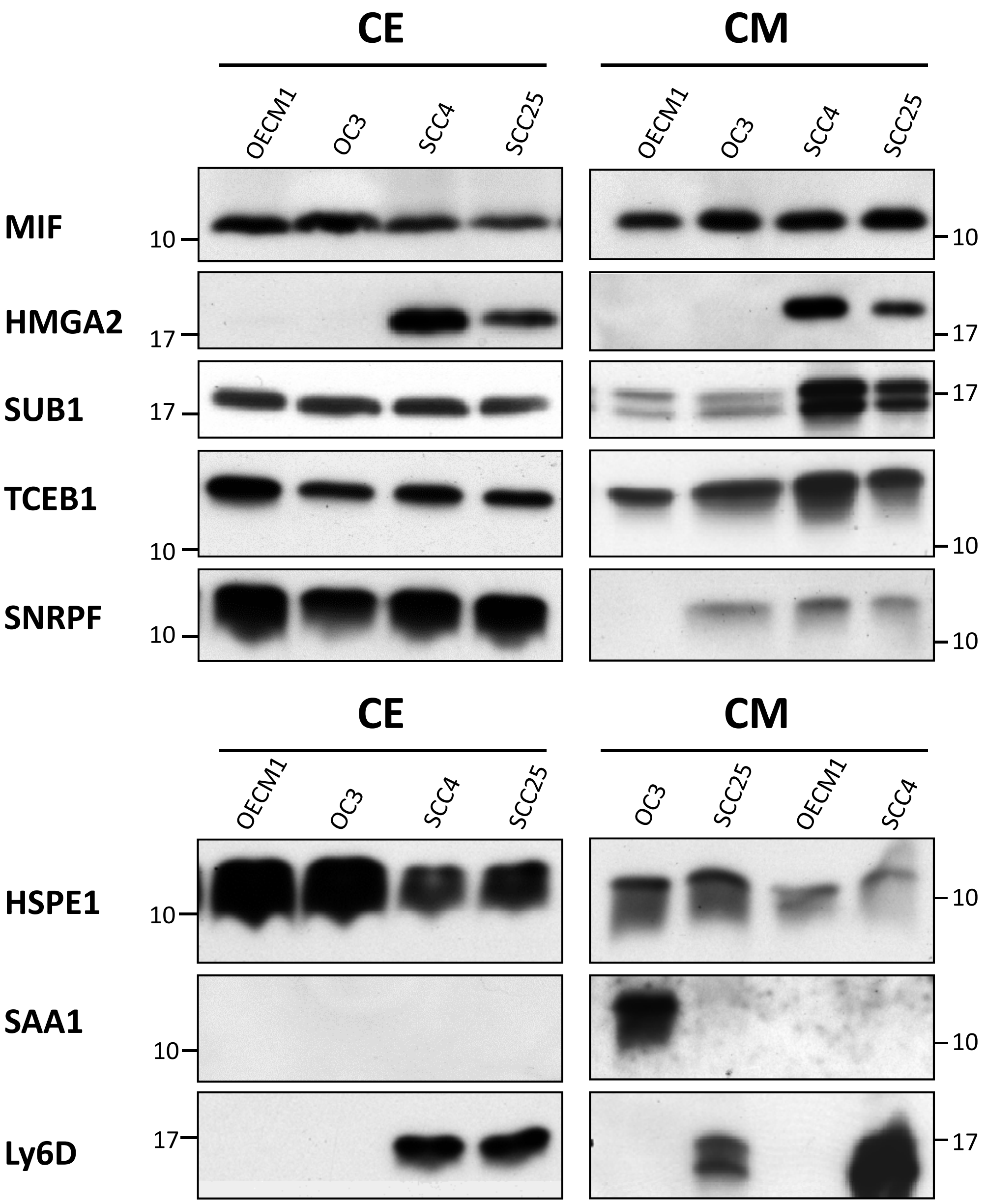

Supplementary Figure 3. Western blot analysis of selected LMr protein targets in cell extracts and conditioned media from OSCC cell lines. Proteins (30 μg) prepared from cell extracts (CE) and conditioned media (CM) of the four OSCC cell lines were separated by 12% SDS-PAGE, transferred to PVDF membranes, and probed with the indicated antibodies, including rabbit polyclonal MIF antibody (Santa Cruz Biotech, Dallas, TX), rabbit monoclonal anti-HMGA2 antibody (Cell Signaling, Danvers, MA), rabbit polyclonal anti-SUB1 antibody (Novus Biologicals, Littleton, CO), mouse monoclonal anti-TECB1 antibody (BD Biosciences, Franklin Lakes, NJ), rabbit polyclonal anti-SNRPF antibody (Proteintech, Chicago, IL), mouse monoclonal anti-HSPE1 antibody (Santa Cruz Biotech, Dallas, TX), mouse monoclonal anti-SAA1 antibody (Abcam, Cambridge, MA), and mouse monoclonal anti-Ly6D antibody (Santa Cruz Biotech, Dallas, TX).

Supplementary Figure 4

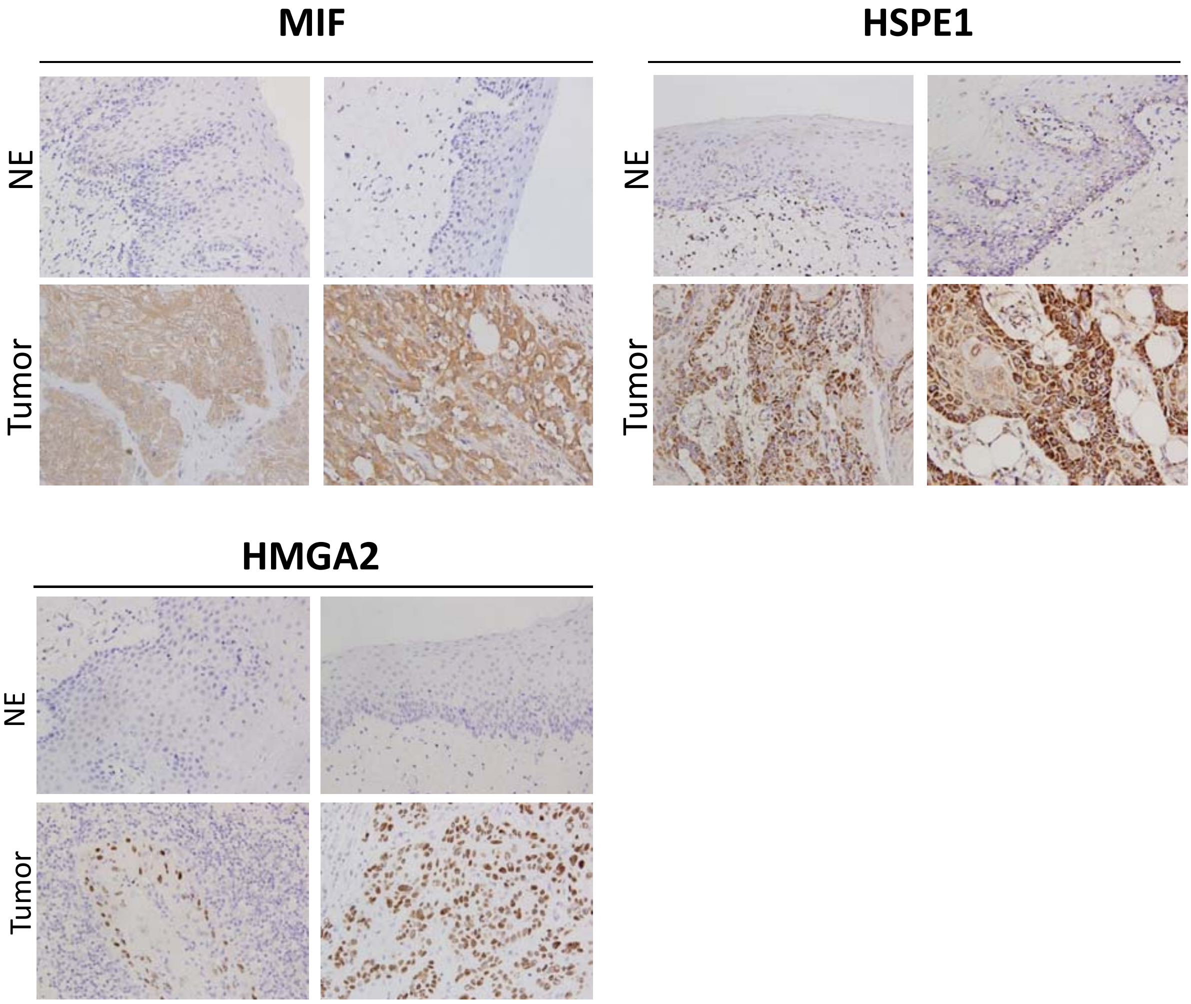

Supplementary Figure 4. Test of applicability of antibodies against MIF, HMGA2 and HSPE1 for immunohistochemical staining of OSCC tissue sections. Shown here are representative images of the stained tissue sections from two OSCC cases for each target. NE, pericancerous adjacent normal epithelia.
